# Schizophrenia Treatment Based on Sustained Release of Risperidone from Poly(lactic-*co*-glycolic) Acid Implantable Microarray Patch

**DOI:** 10.1021/acsami.4c20010

**Published:** 2025-03-06

**Authors:** Linlin Li, Li Zhao, Mingshan Li, Yushi Tao, Akmal Hidayat Bin Sabri, Natalia Moreno-Castellanos, Rand Ghanma, Brett Greer, Qonita Kurnia Anjani, Helen O. McCarthy, Ryan F. Donnelly, Eneko Larrañeta

**Affiliations:** 1 School of Pharmacy, Queen’s University Belfast, 97 Lisburn Road, Belfast BT9 7BL, United Kingdom; 2 CICTA, Department of Basic Sciences, Medicine School, Health Faculty, Universidad Industrial de Santander, Cra 27 calle 9, Bucaramanga 680002, Colombia; 3 Department of Pharmaceutical Technology, Faculty of Pharmacy, Jordan University of Science and Technology, P.O.Box 3030, Irbid 22110, Jordan; 4 Institute for Global Food Security, School of Biological Science, Queen’s University Belfast, 19 Chlorine Gardens, Belfast BT9 5DL, U.K.

**Keywords:** schizophrenia, risperidone, transdermal, implantable, microarray patch

## Abstract

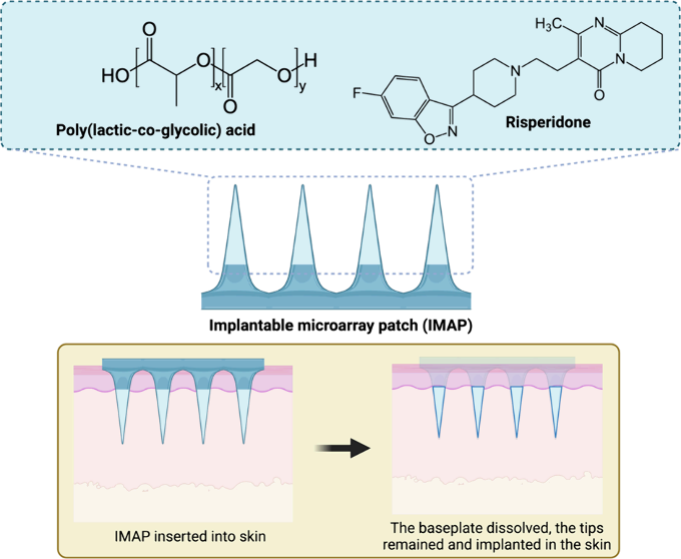

Schizophrenia is one of the most severe mental disorders, affecting approximately 24 million people worldwide. Conventional treatments, such as drug-loaded implants and intramuscular injections, have several limitations, including pain during administration and the need for medical professionals to perform the procedure. In this study, a poly(lactic-*co*-glycolic) acid (PLGA)-based implantable microneedle patch (IMN) was developed for the transdermal delivery of risperidone (RIS) as a treatment for schizophrenia. RIS IMNs were prepared by sequentially casting gel-based formulations into microneedle (MN) molds. The patches were then characterized using microscopy, differential scanning calorimetry, and infrared spectroscopy, as well as through evaluations of MN insertion and RIS release. A selected formulation was further tested by evaluating its cytocompatibility and its ability to deliver RIS in a rat animal model. The RIS IMN demonstrated excellent mechanical properties, successfully inserting up to 378 nm into model skin, which is crucial for effective transdermal drug delivery. A biocompatibility study using human dermal fibroblasts showed no cytotoxicity, with cell viability and proliferation being close to 100%. The optimized formulation achieved a sustained *in vitro* release over 7 days, while *ex vivo* skin deposition and permeation studies showed over 65% RIS delivery efficiency. *In vivo* animal studies confirmed that RIS IMNs maintained therapeutic plasma concentrations throughout the nine-day experiment, with *C*_max_ values of RIS and 9-OH RIS reaching 387.96 ± 194.02 and 139.89 ± 47.68 ng/mL at 6 and 96 h, respectively. In contrast, intramuscular injection showed a *C*_max_ of 1756.70 ± 246.06 and 1377.38 ± 160.78 ng/mL at 2 and 6 h but lost therapeutic effect after just 24 h. These findings suggest that RIS IMNs offer significant clinical benefits for patients with schizophrenia, providing prolonged therapeutic effects with a simple, self-administering drug delivery system, reducing the need for frequent medical interventions.

## 1 Introduction

Schizophrenia is a chronic, severe mental disorder characterized by disturbances in thought processes, perception, emotional responsiveness, and social interactions.^1^ Affecting approximately 24 million people worldwide, it presents a significant public health challenge.^1^ Moreover, individuals with schizophrenia have a reduced life expectancy due to higher risks of suicide, cardiovascular diseases, and inadequate access to medical care.^2^ Therefore, effective management is critical to reduce relapses, hospitalisations, and the overall societal impact of the disease.^3^

One of the most commonly used medications to treat schizophrenia is risperidone (RIS), which is an atypical antipsychotic drug that works by modulating dopamine and serotonin receptors in the brain.^4^ RIS is available in various formulations, including oral tablets, orally disintegrating tablets, and long-acting injectable (LAI) formulations.^5^ The oral formulations are typically administered daily, while the LAI versions, such as Risperdal Consta and Perseris, provide sustained drug release over two to 4 weeks, offering improved treatment adherence by reducing the need for daily administration.^6^

Despite their effectiveness, current RIS formulations have several limitations. Oral RIS requires strict adherence to daily dosing, which can be difficult for patients with schizophrenia, leading to poor compliance and increased risk of relapse.^7−9^ For example, in the short term, 41.7% of patients do not stick to the prescribed treatment whereas in the long run the number is even higher.^10^ Although LAI formulations address this by offering extended drug release, they still require frequent clinic visits for injections, posing logistical challenges and discomfort for patients. Additionally, the invasive nature of injections, combined with potential side effects, such as injection site reactions and sedation, highlights the need for alternative drug delivery systems that offer both sustained release and enhanced patient comfort.

Microneedle (MN) arrays represent an innovative transdermal drug delivery approach, offering a noninvasive and patient-friendly alternative to traditional methods such as injections or oral medications.^11−14^ MNs consist of tiny projections that painlessly penetrate the outer layer of the skin, delivering drugs directly into the dermal tissue for systemic absorption or localized treatment.^15−18^ This method bypasses gastrointestinal metabolism and enhances bioavailability, making it ideal for delivering a wide range of therapeutic agents, including small molecules, proteins, and vaccines.^19^ There are five primary types of microarray patches: dissolving MNs, which dissolve in the skin to release their drug payload; hydrogel-forming MNs, which absorb interstitial fluid and swell to deliver the drug; coated MNs, which are coated with a drug formulation and release the drug upon insertion; hollow MNs, which deliver liquid formulations through a hollow channel; and solid MNs, which create microchannels in the skin but require the drug to be delivered through another route, often as a subsequent topical application.^19^ Each type of MNs has unique advantages and applications, making them promising candidates for improving patient compliance and enhancing the efficacy of various therapies.

Recently, MNs capable of providing long-term drug release have drawn increasing attention.^20−31^ One of the strategies to achieve this is to use implantable MNs (IMNs) containing drug loaded micro implants based on biodegradable polymers such as poly(lactic-*co*-glycolic) acid (PLGA). Unlike the five types of MNs that provide immediate drug release, IMNs are designed for sustained delivery over extended periods. IMNs can be formulated with various materials, including biodegradable polymers like PLGA, which allow for gradual drug release as the polymer degrades.^32,33^ This controlled release minimizes the peaks and troughs often associated with conventional dosing regimens, enhancing therapeutic efficacy and patient adherence. The sustained-release characteristic is particularly advantageous for administering medications that require steady plasma concentrations, such as antipsychotics, analgesics, or hormones, including RIS. Moreover, the minimally invasive nature of IMNs reduces discomfort and the risk of complications compared to injections.^12^ Overall, IMNs represent a promising advancement in drug delivery, potentially improving treatment outcomes for patients with chronic conditions by enabling self-administration, reducing the need for frequent medical visits, and providing prolonged therapeutic effects.

In this study, a novel biocompatible IMN for delivering RIS was developed. Mechanical and insertion performances were measured. *In vitro* and *ex vivo* drug release studies were conducted to establish relevant evidence before carrying out the *in vivo* animal study. The RIS IMN utilizes drug loaded PLGA MNs to implant the medication into the skin, enabling a sustained release of the drug over an extended period while maintaining stable plasma concentrations.

## 2 Materials and Methods

### 2.1 Materials

Risperidone (RIS) was resourced from Enke Pharma-Tech Co., Ltd. (Cangzhou, China). Viatel DLG 5005E poly(d,l-lactide-*co*-glycolide) (PLGA) was purchased from Ashland (Dublin, Ireland) while Plasdone K-29/32 (poly(vinylpyrrolidone), PVP, MW 58 kDa) was donated by Ashland (Kidderminster, U.K.). Poly(vinyl alcohol) (PVA, MW 31–50 kDa, 87%–89% hydrolyzed), poly(vinyl alcohol) (PVA, MW 9–10 kDa), poly(ethylene glycol) (PEG) (average MW 400, and 600 Da), sodium lauryl sulfate (SLS), phosphate buffered saline (PBS) tablets (pH 7.4), HPLC-grade acetonitrile (ACN), methanol (MeOH), dichloromethane (DCM), and triethylamine (TEA) were all purchased from Sigma-Aldrich, (Dorset, U.K.). Dimethyl sulfoxide (DMSO) was obtained from VWR International Limited (Leicestershire, U.K.) while phosphoric acid (purity 85%) was provided by Fluorochem Limited (Hadfield, U.K.). Deionized water was produced by an ELGA purified PureLab water purification system (High Wycombe, U.K.).

### 2.2 Fabrication of RIS IMNs

**Figure 1:**
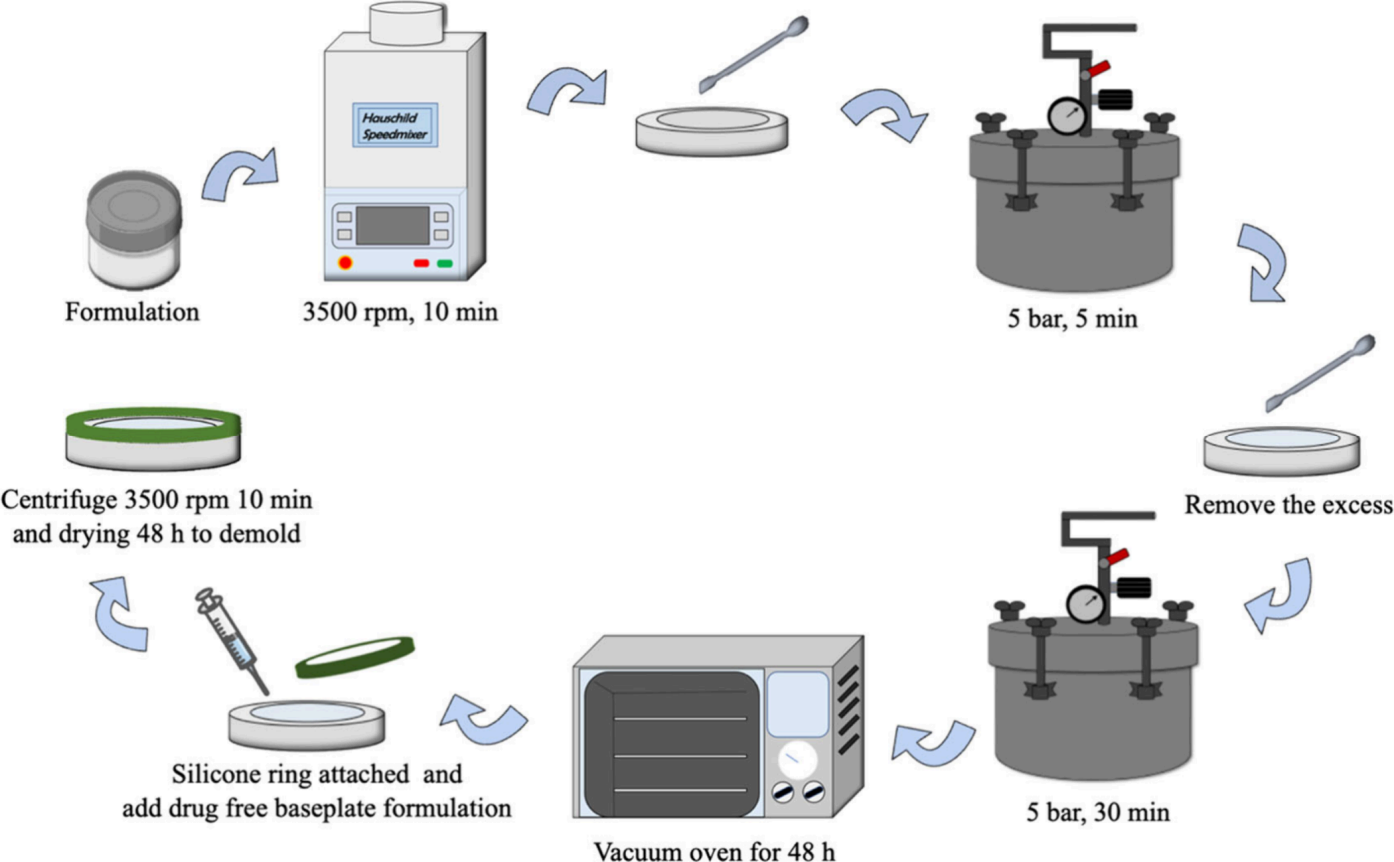
Schematic presentation and summary of the fabrication process of the RIS IMN.

Polydimethylsiloxane molds with a 16 × 16 array of pyramidal structures was used to prepare the RIS PLGA IMN. The pyramidal tip length measured 600 μm, with a cuboidal base column of 250 μm, and the base width was 300 μm. Each MN patch covered a total area of 0.36 cm^2^. The fabrication steps are outlined in Figure 1. The process began by casting the tips of the MNs, where formulations were prepared by dissolving ingredients in DMSO or DCM. These mixtures were then homogenized using a Speedmixer DAC 150 FVZ-K (Synergy Devices Ltd., London, U.K.) for 10 min at 3500 rpm, with zirconium beads included as milling media. The formulations were then applied to the MN molds and placed in a Protima pressure tank AT10 (Richmond Scientific, Chorley, U.K.) at 5 bar for 5 min to ensure the cavities were filled. Excess material was removed from the molds using a spatula before they were returned to the pressure tank for an additional 30 min to ensure complete filling of the tip section. Afterward, the molds were placed in a vacuum oven to facilitate removal of the organic solvent.

The second step, which involved casting the baseplate, was performed after the tip layer had completely dried. The baseplate was formulated using a mixture of 30% w/w PVA (31–50 kDa) and 40% w/w PVP (58 kDa) in a 1:1 ratio. Rings were attached to the surface of the molds to contain the baseplate formulation, which was added using a positive displacement pipet and centrifuged at 3500 rpm for 10 min to fill the cavities. The systems were left for 24 h to allow the water to evaporate. Once dried, the RIS PLGA MNs were peeled off and placed in an oven at 37 °C to ensure they dried completely. All MNs were stored in sealed 24-well plates within a vacuum desiccator until further use.

### 2.3 Evaluation of the Mechanical Properties of RIS IMNs

To assess the mechanical properties of the RIS IMNs, a TA-XT2 texture analyzer (Stable Microsystems, Haslemere, U.K.) was employed in compression mode. The initial heights of the microneedles were first measured and documented using a Leica EZ4D Digital Microscope (Leica Microsystems, Milton Keynes, U.K.). Each MN was then affixed to a movable aluminum probe with double-sided sticky tape and lowered at a rate of 0.5 mm/s until it was compressed against an aluminum block with a force of 32 N, simulating the force typically applied during manual skin application. The compression was maintained for 30 s. Afterward, the compressed MNs were examined again under the digital microscope to record the postcompression height. The percentage of height reduction was then calculated using eq 1.
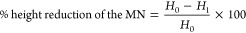
1where *H*_0_ is the height of MNs before compression and *H*_1_ is the height of MNs after compression. All measurements were repeated 8 times (*n* = 8).

### 2.4 Insertion Study of RIS IMNs

To assess the insertion capability of the RIS IMNs, Parafilm M was employed as a skin simulant, as validated by our previous studies.^34^ A total of eight layers of Parafilm M were stacked, forming a film roughly 1 mm thick, with each individual layer being 126 μm.^34^ The insertion test was conducted using the texture analyzer. Each MN was first attached to a movable aluminum probe, which was then lowered onto the film at a speed of 0.5 mm/s until a force of 32 N was applied. This force was held constant for 30 s before retraction. Afterward, the film was unfolded and each layer was inspected under a digital microscope to count the number of holes formed. The percentage of MNs that successfully penetrated each layer was calculated using eq 2. All tests were conducted in quadruplicate (*n* = 4).
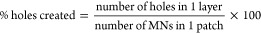
2

### 2.5 Differential Scanning Calorimetry Analysis

In order to investigate potential interactions and assess the degree of crystallinity between the drug and excipients, differential scanning calorimetry (DSC) (DSC Q100, TA Instruments, New Castle, Delaware, USA) was performed on the components of the formulation including RIS powder, PLGA, and the dry powder from the F2 formulation. A measured sample (5–10 mg) was carefully weighed and placed into a DSC aluminum pan, which was covered with an aluminum lid using a crimping method. An empty, sealed aluminum pan served as a reference to enable baseline calibration. The samples were then heated from room temperature to 250 °C at a rate of 10 °C/min. The resulting thermograms were then recorded and analyzed.

### 2.6 Attenuated Total Reflectance Fourier Transform Infrared Analysis

ATR-FTIR spectroscopy (Accutrac FT/IR-4100 Series, Jasco, Essex, U.K.) was also employed to further explore potential chemical interactions between the drug and excipients. Spectra were captured in the range of 4000 to 600 cm^–1^ at room temperature, with a resolution of 4 cm^–1^, averaging 32 scans to produce each spectrum.

### 2.7 RIS Saturation Solubility Study

To determine the saturation solubility of RIS in various solvents, including PBS (pH 7.4) and different concentrations of methanol, PEG 400, PEG 600, Tween 80, and SLS in PBS, an excess amount of RIS was added to glass vials containing 5 mL of each solvent. Full saturation was ensured by placing the vials in an orbital incubator set at 37 °C and 100 rpm for 24 h. After incubation, the saturated RIS solutions were filtered through 0.2 μm syringe filters to remove any undissolved drug. The filtered samples were then diluted with methanol and analyzed using HPLC. Each measurement was conducted in quadruplicate (*n* = 4).

### 2.8 Drug Content Analysis of RIS IMNs

To assess the drug content, each RIS IMN was first placed in a 5 mL glass vial containing 1 mL of water and sonicated in a water bath for 1 h to remove the water-soluble baseplate layer. Following this, 4 mL of DMSO was added to dissolve RIS and PLGA. Once fully dissolved, the solution was diluted with methanol and filtered through 0.2 μm syringe filters. The samples were then analyzed using HPLC with six replicates (*n* = 6).

### 2.9 *In Vitro* Release Study of RIS IMNs

To determine the *in vitro* release profiles of RIS IMNs, agarose gel and a Franz cell apparatus were employed. Agarose gel with a concentration of 2% (w/w) was prepared by dissolving agarose powder in the release medium via microwave heating. The solution was then poured into a Petri dish and allowed to cool and subsequently gel. A 1% w/v SLS solution in PBS, used as the release medium to maintain sink conditions, was also prepared. In a typical application, one agarose gel film was secured to the donor compartment with cyanoacrylate glue, and a RIS IMN was manually inserted and held in place for 30 s. The donor compartment was then mounted onto the receiver compartment, which contained 12 mL of the release medium. The temperature of the receiver compartment was maintained at 37 °C with a magnetic stir bar stirring at 600 rpm. To minimize solvent evaporation, the donor chamber and sampling port were sealed with Parafilm M throughout the experiment. At prespecified time intervals, 200 μL samples were withdrawn and replaced with an equal volume of fresh release medium. Sampling times included 1, 2, 4, 6, and 8 h on the first day, and then once daily until day 7. On day 7, the system was dismantled. Samples collected were diluted with methanol, filtered through 0.2 μm syringe filters, and analyzed using the HPLC method. All measurements were conducted in quadruplicate (*n* = 4).

### 2.10 *Ex Vivo* Skin Deposition and Permeation Study of RIS IMNs

The *ex vivo* study of skin deposition and permeation for RIS IMN was conducted using excised full-thickness porcine skin and a Franz cell. Full-thickness porcine skin was obtained from stillborn piglets, which were frozen at −20 °C before use. Prior to skin removal, the piglets were defrosted for 24 h. The skin was then carefully excised using a disposable scalpel and shaved with a disposable razor. For the release medium, PBS containing 1% w/v SLS was prepared to maintain sink conditions, and the porcine skin was equilibrated in this medium for 30 min. After equilibration, the skin was blotted with tissue paper to remove excess aqueous medium and attached to the donor compartment by using cyanoacrylate glue. Each RIS IMN was then manually inserted into the porcine skin and held in place for 30 s. A stainless-steel weight was placed on the baseplate of each RIS IMN to secure it. The donor compartment was then mounted onto the receiver compartment, which contained 12 mL of the release medium. The temperature of the receiver compartment was maintained at 37 °C throughout the experiment with a magnetic stir bar stirring at 600 rpm. To prevent evaporation, the donor chamber and sampling port were sealed with Parafilm M.

Samples of 200 μL were collected at intervals of 1, 2, 4, 6, 8, and 24 h and replaced with fresh release medium. The whole setup was disassembled after 24 h. The porcine skin was then removed from the donor compartment and gently wiped to eliminate any residual baseplate. The area of skin where the RIS IMN had been applied was cut into small pieces and placed in an Eppendorf tube with 0.5 mL of deionized water and two metal beads. The tubes were processed in a Tissue Lyser LT (Qiagen, Ltd., Manchester, U.K.) for 30 min at 50 Hz to break down and dissolve the residual polymers in the skin. Following this, 1 mL of DMSO was added to each tube and mixed for an additional 30 min to extract RIS and dissolve PLGA. The samples were then rinsed with 3.5 mL of DMSO, transferred to glass vials, and sonicated in a water bath for 30 min. The resultant samples from the porcine skin and those collected at the specified intervals were diluted with methanol, filtered through 0.2 μm syringe filters, and analyzed using HPLC. All measurements were conducted in quadruplicate (*n* = 4).

### 2.11 Biocompatibility Studies of RIS IMNs

To assess the cytotoxic effect of RIS IMN on human dermal fibroblast cells (HDFa), cell viability and proliferation were evaluated using MTT, 3-(4,5-dimethylthiazol-2-yl)2,5-diphenyltetrazolium bromide (MTT, Sigma-Aldrich, St. Louis, MO, USA), live/dead, and PicoGreen assays. In brief, HDF cells (5 × 10^3^ cells/well) were cultured in DMEM (Sigma-Aldrich, Saint Louis, USA) supplemented with 10% fetal bovine serum (FBS) and 1% penicillin–streptomycin solution (Sigma-Aldrich, Saint Louis, USA) in 48-well plates for 48 h. For cell viability, the MTT assay was performed by adding an MTT solution (5 mg/mL) to each well and incubating the cells for 5 h at 37 °C in a 5% CO_2_ environment. After incubation, the cells were washed with PBS, and DMSO was added to dissolve the formazan crystals. The absorbance was measured at 570 nm by using a Multiskan GO spectrophotometer (Thermo Fisher Scientific, USA). To further validate the MTT results, a live/dead assay was conducted. Cells, both treated and untreated with the formulations, were cultured for 48 h and then incubated with Calcein AM and ethidium homodimer-1 (Thermo Fisher Scientific, Wilmington, DE, USA) for 30 min at room temperature. Fluorescence imaging was performed using an Olympus BX53 fluorescence microscope (Olympus America Inc., NY, USA), and the images were processed using ImageJ software (NIH, USA). For evaluating cell proliferation, the PicoGreen assay was performed to measure DNA synthesis during the cell cycle. A calibration curve was used to determine the DNA concentrations. Cells, both treated and untreated with the formulations, were incubated for 48 h, then frozen at −80 °C in DNA lysis water. TE buffer and a PicoGreen solution were subsequently added. Control cells cultured on plates were used for comparison. The samples were agitated for 2 min and incubated in the dark at room temperature for 8 min. Fluorescence was measured at 480 nm excitation and 520 nm emission by using a Varioskan Flash plate reader (Thermo Fisher Scientific, USA).

### 2.12 *In Vivo* Drug Delivery of RIS IMNs

The *in vivo* pharmacokinetic study of RIS IMN was conducted in alignment with the guidelines of the Federation of European Laboratory Animal Science Associations (FELASA) and the European Convention for the Protection of Vertebrate Animals, following the principles of the 3Rs: replacement, reduction, and refinement. Ethical approval for the study was granted by the Committee of the Biological Services Unit at Queen’s University Belfast, under License number PPL 2903, with personal license numbers PIL 2127, 2205, and 2206.

In this study, 16 healthy female Sprague-Dawley rats (aged 10–12 weeks, weighing 315 ± 22.31 g) were divided into two groups: an RIS IMN group (test group) and an intramuscular injection (IM) group (control group), as detailed in Table 1. Each rat in the RIS IMN group received four IMNs, while rats in the IM group were administered RIS nanosuspensions intramuscularly. The dosage of RIS was based on prior studies, with the IMN group receiving 20.32 mg/kg and the IM group receiving 12.22 mg/kg.^35^

**Table 1:** Details of Doses Plan for Each Cohort in the Animal Study

cohort	number of rats	dosing method	total dose administered (mg/kg)	total dose administered (mg/rat)
MN	8	4 MN patches per rat	20.32	6.4
IM	8	100 μL of RIS nanosuspension per rat	12.22	3.85

To prepare the RIS nanosuspension for IM injection, the wet-milling technique was utilized. The process began by combining 200 mg of RIS powder with 3.5 mL of 2% w/w PVA (9–10 kDa) as a polymeric stabilizer. Two cylindrical magnetic stirring bars and 2 mL of 0.1–0.2 mm diameter ceramic beads were also added to the glass vial, and the mixture was agitated on a magnetic stirrer plate at 1500 rpm for 24 h. Following agitation, the RIS nanosuspensions were separated from the ceramic beads and magnetic bars by passing the mixture through a 200-mesh nylon sieve. The beads were then rinsed with 5 mL of water to maximize the yield of the drug. Next, 3.5 mL of 2% w/w PVP (58 kDa) was added to the nanosuspensions as a cryoprotectant. The samples were prefrozen at −80 °C for 1 h before undergoing freeze-drying, following a previously developed protocol.^36^ To prepare the formulation for IM injection, 65.45 mg of the lyophilized RIS nanosuspensions was dissolved in 934.55 μL of sterilized water. From this solution, 100 μL of the resuspended nanosuspensions was injected into each rat for the *in vivo* study.

To minimize interference from hair during the experiment, the dorsal hair of the rats in the MN cohort was removed 1 day prior to the experiment. The hair on their backs was first trimmed by using electric clippers. To ensure complete removal, hair removal cream was applied to the shaved area for 10–15 min. This process ensured a smooth skin surface for the application of MNs during the study. The entire process of hair removal was conducted under anesthetic conditions.

**Figure 2:**
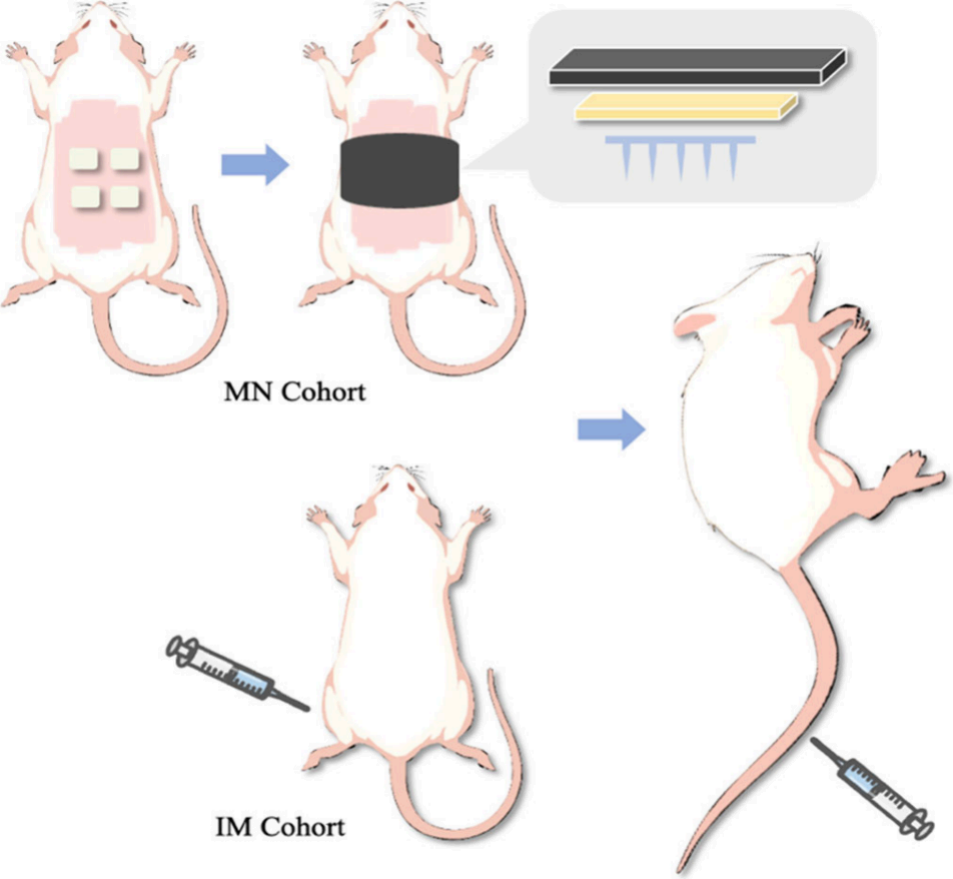
Schematic presentation of dosage administration and samples taken of two cohorts.

On the following day, after being sedated again with gaseous anesthesia (2–4% v/v isoflurane in oxygen), RIS IMN were applied to the backs of the rats in the IMN cohort (*n* = 8). Each IMN was manually inserted into the skin and held for 30 s with firm pressure. A total of four patches were applied to each rat. To ensure the patches stayed in place during the course of experiment, Microfoam surgical tape (3M, Bracknell, U.K.) was used to cover the patches, and kinesiology tape was gently wrapped around the rats’ backs and abdomens. The rats were housed individually for 24 h to prevent them from disturbing or consuming the IMNs. For the IM cohort (*n* = 8), each rat received an injection of 100 μL of a RIS nanosuspension in the thigh. The process of RIS IM injection and IMN application is illustrated in Figure 2. After 24 h, all the RIS IMNs were removed from the backs of the rats under gaseous anesthesia. Upon completion of the *in vivo* study on day 10, all rats were euthanised using a CO_2_ chamber.

According to the university’s Institutional Project License, each rat was only allowed to be bled twice within a 24 h period and no more than 10 times in a month. As a result, blood samples were taken from 4 rats in each cohort at the 1 and 4 h marks and from the other 4 rats at the 2 and 6 h marks. All 16 rats were sampled at the 24 h point and then at various intervals for up to 9 days. Specifically, blood samples were collected at the following predetermined time points: 1, 2, and 4 h (4 rats at each point) and then at 1, 2, 3, 4, 6, and 9 days (8 rats at each time point). The samples were taken via tail prick bleed using preheparinized 23G butterfly needles and collected into preheparinized Eppendorf tubes. The collected samples were immediately centrifuged at 2200*g* for 10 min at 4 °C in a temperature-controlled centrifuge to separate the plasma from the blood. The plasma samples were then transferred into 0.5 mL polypropylene tubes and stored at −20 °C until further analysis.

To extract RIS and its active metabolite paliperidone (9-OH RIS) from the plasma samples, 0.5 mL of ethyl acetate was added to each sample. These mixtures were vortexed (Vortex, Fisons Scientific Equipment, Loughborough, Leicestershire, U.K.) at 1500 rpm for 25 min to facilitate the extraction of the drugs from the plasma. Following this, plasma proteins were precipitated by centrifuging the samples at 14 000*g* for 10 min at room temperature. The supernatant, which contained the extracted drugs, was carefully removed and placed in glass culture tubes. The solvent (ethyl acetate) was then evaporated using a Zymark TurboVap LV Evaporator Workstation, set to 40 °C, with a stream of nitrogen gas applied at 5 psi for 20 min. After evaporation, the remaining residue was reconstituted in 50 μL of ACN and vortexed for 1 min to ensure the drug extracted was completely dissolved. The reconstituted samples were transferred to glass inserts with polymeric feet inside Agilent vials, ready for analysis. A liquid chromatography–tandem mass spectrometry (LC–MS/MS) system was used to quantify the RIS and 9-OH RIS concentrations, and the data were processed using Mass Lynx software for accurate analysis.

### 2.13 Pharmaceutical Analysis

All RIS quantifications were carried out using an Agilent 1200 reverse phase high-performance liquid chromatography (RP-HPLC) system (Agilent Technologies UK Ltd., Stockport, U.K.) equipped with a UV detector set to 280 nm and a Phenomenex C18 ODS column (250 mm in length, 4.60 mm internal diameter, and 5 μm particle size). The mobile phase consisted of a 20% v/v aqueous solution (1% TEA, pH 6.2), 20% v/v acetonitrile (ACN), and 60% v/v methanol. The injection volume was 40 μL, with a column temperature of 25 °C and a flow rate of 1 mL/min. The retention time for RIS detection was recorded at 4.8 min.

For simultaneous RIS and 9-OH RIS analysis, an Acquity UPLC i-Class system was used, incorporating a column manager, binary system, and sample manager, all linked to a Xevo TQ-MS (triple quadrupole MS/MS) mass spectrometer (Waters, Manchester, U.K.) with an electrospray ionization (ESI) source. Both analytes were identified in positive electrospray mode. The separation and quantification of RIS and 9-OH RIS were achieved using a Waters Symmetry C18 column (150 mm in length, 4.60 mm internal diameter, 5 μm particle size). The column temperature was kept at 35 °C. The mobile phase was isocratic and consisted of acetonitrile and 10 mM ammonium formate (80:20), with a flow rate of 0.5 mL/min. Each sample injection volume was 5 μL, and the total run time was 4 min. Data were processed using the Mass Lynx software, with retention times for RIS and 9-OH RIS at 2.64 and 2.56 min, respectively.

### 2.14 Data Analysis

The data were processed using Microsoft Excel (Redmond, WA, USA), with results presented as mean values ± standard deviation. Statistical and pharmacokinetic analyses were performed using GraphPad Prism version 10.2.0 (GraphPad Software, San Diego, CA, USA). For comparisons between any two groups, a *t* test was applied, while one-way ANOVA was used for analyzing data from more than two groups. A *p*-value of less than 0.05 was considered statistically significant in all analyses.

## 3 Results and Discussion

### 3.1 Fabrication of RIS IMNs

The formulations used for preparing RIS IMNs are detailed in Table 2. Basically, the RIS IMNs were fabricated using a two-step casting approach, which localized RIS in the tip part of the MNs. Since the MN tips cannot be fully dissolved into the skin compared with dissolving MNs that contain drug in the entire needles, this method reduces drug wastage and improves delivery efficiency.^37,38^ The tip part of the MNs consisted of RIS and PLGA. PLGA is one of the most commonly used biodegradable synthetic polymers for medical applications.^39,40^ The degradation of PLGA occurs through hydrolysis, where water hydrolyses the ester linkages in PLGA, causing it to degrade.^41^ It is well established that 50:50 PLGA degrades in 1–2 months.^42^ PVA (31–50 kDa) and PVP (58 kDa) were selected to fabricate the baseplate of MN patches in this study. They are both safe, inexpensive, biocompatible, biodegradable, and considered nontoxic by the USA FDA.^43^ Research has shown that the combination of PVA and PVP can guarantee high mechanical strength of MNs.^44^ Additionally, they can both be rapidly eliminated from the body due to their high water solubility and low molecular weight (lower than 60 kDa).^26^

**Table 2:** Formulations Containing RIS for the Fabrication of RIS IMN

formulation code	RIS % (w/w)	PLGA 5005E % (w/w)	solvent type	solvent % (w/w)	casting times
F1	8.0	32.0	DMSO	60.0	1
F2	16.0	24.0	DMSO	60.0	1
F3	24.0	16.0	DMSO	60.0	1
F4	26.7	40.0	DMSO	33.3	1
F5	20.0	30.0	DMSO	50.0	1
F6	25.0	25.0	DCM	50.0	1
F7	16.0	24.0	DMSO	60.0	2

Table 2 summarizes all the formulations used to prepare RIS IMNs, which were fabricated using a two-step casting method. It helps concentrate the drug in the tip of the MNs, thus reducing potential drug wastage and increasing drug delivery efficiency. PLGA was used in this case as it is one of the only few FDA-approved biodegradable biomedical polymers and has been widely used to fabricate MNs for sustained released of small molecules.^22,25^

In total, seven formulations with different percentages of RIS, PLGA, and solvent as well as different casting times and different choices of solvent were used to prepare RIS IMNs. Table 3 summarizes morphologies of the resulting RIS IMNs if the respective formulation led to well-formed IMNs as well as the appearances of the drug/polymer mixtures which could not be used to prepare IMNs. As can be seen, RIS IMNs prepared from formulations F1, F2, F3, and F7 (RIS-IMN-F1, RIS-IMN-F2, RIS-IMN-F3, RIS-IMN-F7) had well-formed structure as well as sharp and homogeneously drug concentrated tips, which are ideal for skin insertion and transdermal drug delivery. Therefore, these four RIS IMNs were taken for further characterisations. In contrast, F4 and F6 were unable to be used for IMN fabrication due to their viscous nature. F4 contained 40% w/w of PLGA but only 33.3% w/w of DMSO, which is not enough to fully dissolve or sufficiently disperse drug/polymer mixture. F6 may contain sufficient amount of solvent. However, DCM has a very low boiling point of 39.6 °C, which makes it extremely easy to evaporate even at room temperature. As a result, the solvent had already completely evaporated after homogenization of the mixture in the speed mixer.

**Table 3:**
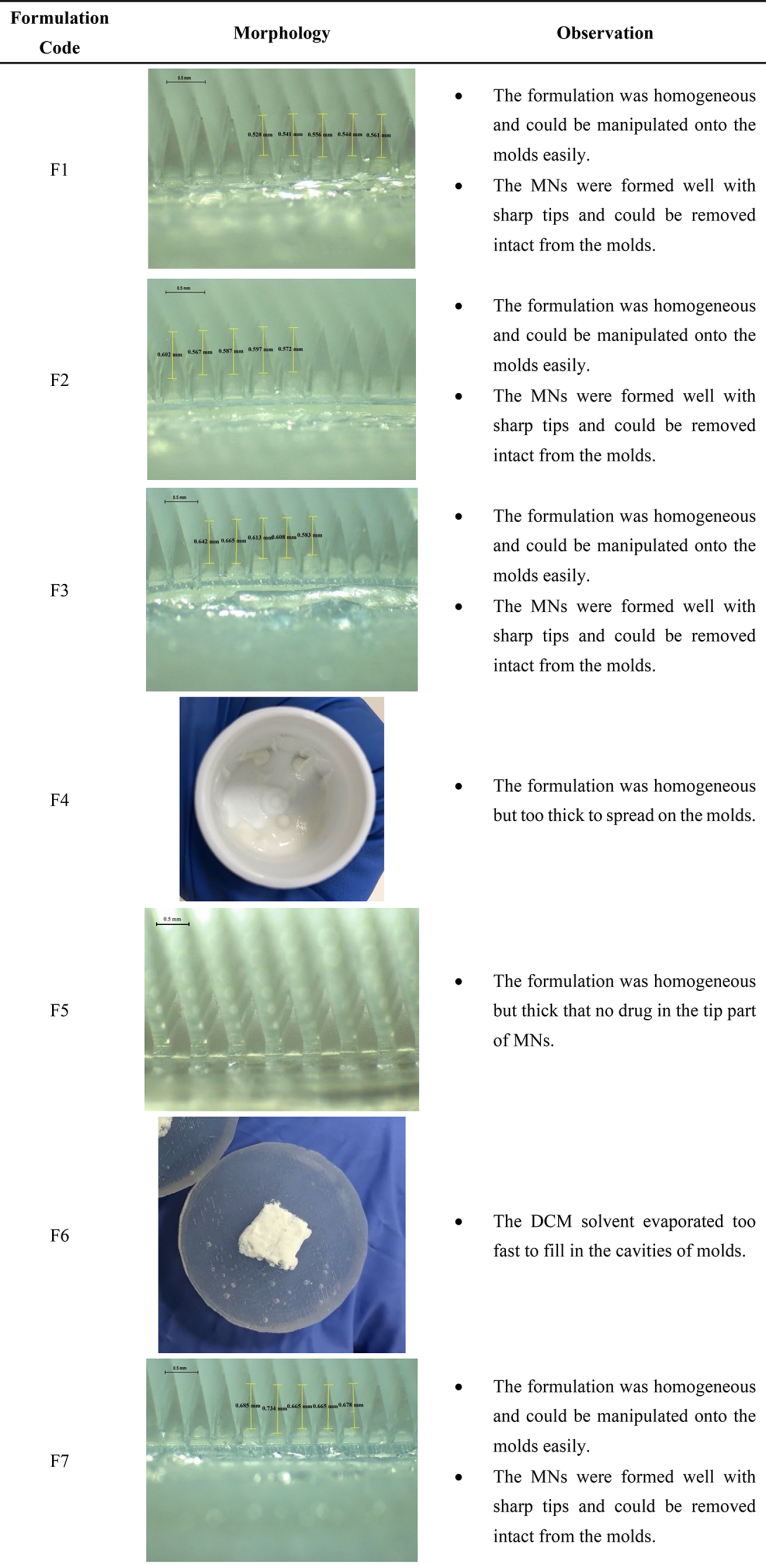
Summary of the Observation of RIS Implantable MN Patches with Different Compositions

### 3.2 Evaluation of the Mechanical Properties of RIS IMNs

**Figure 3:**
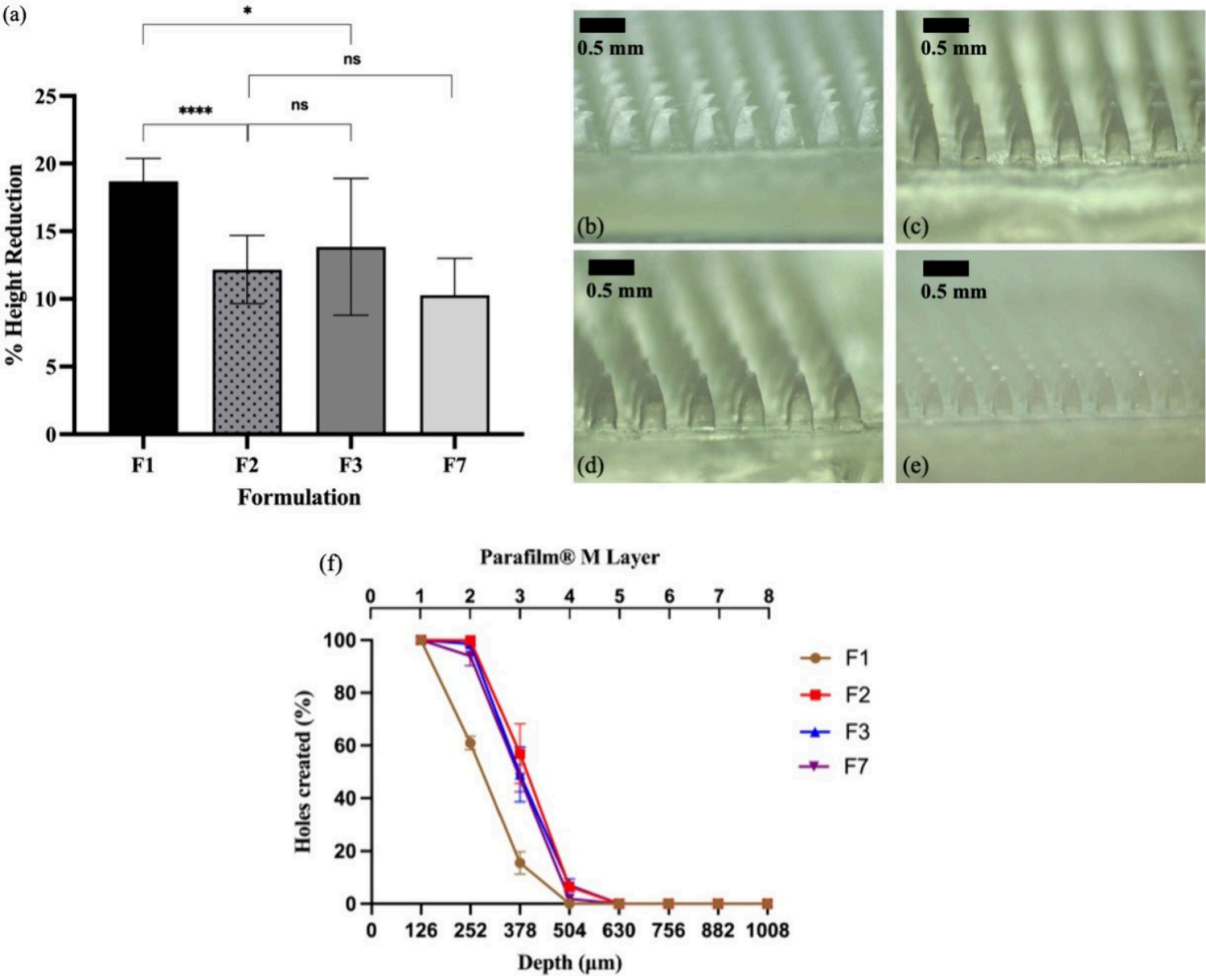
(a) Mechanical properties of three formulations using 32 N per patch compression (mean values ± sd, *n* = 8; *p < 0.05, ****p < 0.0001). Microscope images of MN patches after compression were obtained: (b) RIS-IMN-F1, (c) RIS-IMN-F2, (d) RIS-IMN-F3, (e) RIS-IMN-F7. Scale bar 0.5 mm. (f) Insertion depth and holes created of three formulations of RIS implantable MN patches in skin simulant model Parafilm M (mean values ± sd, *n* = 4).

The outmost skin layer, *stratum corneum*, is a rigid layer formed by dead tissue that protects the human body from the environment. Insufficient mechanical strength of MNs may lead to MN tip breakage during insertion, which may also cause injury to surrounding tissues.^45^ In order to achieve a successful transdermal drug delivery, MNs need to possess sufficient mechanical strength in order for the MN tips to pierce through the *stratum corneum* into the deep epidermis before releasing therapeutic cargos. Therefore, four RIS IMNs (RIS-IMN-F1, RIS-IMN-F2, RIS-IMN-F3, RIS-IMN-F7) were evaluated for their mechanical properties. The percentage of height reduction of four RIS IMNs and the images showing them after mechanical compression are presented in Figure 3. A force of 32 N was used for compression as it is deemed equivalent to the force an average person uses for manual skin insertion.^34^

As can be seen in Figure 3a, the percentages of height reduction were 18.69 ± 1.69%, 12.17 ± 2.51%, 13.84 ± 5.06%, and 10.28 ± 2.70% for RIS-IMN-F1, RIS-IMN-F2, RIS-IMN-F3, and RIS-IMN-F7, respectively. Height reduction was significantly more in RIS-IMN-F1 than in three other RIS IMNs (*p* < 0.0001 between RIS-IMN-F1 and RIS-IMN-F2, *p* < 0.05 between RIS-IMN-F1 and RIS-IMN-F3), whose heigh reductions had no significant differences (*p* = 0.4151 between RIS-IMN-F2 and RIS-IMN-F3, *p* = 0.1707 between RIS-IMN-F2 and RIS-IMN-F7). The most distinct difference between RIS-IMN-F1 and the other three RIS-IMNs is that the percentage of PLGA in the formulation is much higher, which is probably the cause of the weak mechanical performance observed here. There are a few reasons contributing to the relatively poor mechanical property of PLGA. First of all, PLGA is an amorphous polymer with its macromolecular chains arranged in a nonorderly manner, hence less intermolecular forces within its macromolecular network, which results in the overall poor mechanical performance.^45^ In addition, PLGA is a copolymer with two of its components having different molecular structures and functional groups, which further decreases its molecular regularity and in turn weakens its mechanical properties. Nevertheless, the height reductions of the other three RIS IMNs are still comparable to IMNs fabricated in previous research where PLGA-based IMNs successfully pierced the skin and delivered therapeutic cargos.^22,25^ Moreover, as can be seen in Figure 3b–e, the MN tips became bent after compression rather than broken away from the MNs, indicating that the RIS loaded PLGA MN tips are not fragile; hence there are no concerns these MN tips would break away upon insertion.

### 3.3 Insertion Study of RIS IMNs

It is important to evaluate the capability of RIS IMNs to pierce through the skin carrier layer. A commonly used approach for MNs is the insertion study. In this work, an 8-layer Parafilm M film, a validated and widely adopted skin model, was used as the skin simulant to evaluate the insertion ability of the RIS IMNs.^34^Figure 3f shows the insertion depth and percentages of holes created by RIS-IMN-F1, RIS-IMN-F2, RIS-IMN-F3, RIS-IMN-F7, all of which were able to penetrate the first layer. Furthermore, only 60.94 ± 2.61% and 15.53 ± 4.25% of the MN tips on RIS-IMN-F1 were able to penetrate the second layer and third layer, respectively, demonstrating poor insertion capability. In contrast, almost all MN tips RIS-IMN-F2, RIS-IMN-F3, and RIS-IMN-F7 were able to penetrate the second layer, with noticeable differences in the number of holes in the third layer. Specifically, 56.84 ± 11.37% of MN tips on RIS-IMN-F2, 49.02 ± 10.40% of MN tips on RIS-IMN-F3, and 47.66 ± 5.22% of MN tips on RIS-IMN-F2 are able to penetrate the third layer. Nevertheless, there is no statistically significant difference between them (*p* > 0.05). These findings align with the mechanical properties study, which showed that F2, F3, and F7 were mechanically stronger than F1, contributing to their superior insertion ability. Previous research also reported similar outcomes.^20^ The thickness of each Parafilm M layer is approximately 126 μm, suggesting that the insertion depth for RIS-IMN-F2, RIS-IMN-F3, RIS-IMN-F7 ranged between 378 and 504 μm.^34^ Given that the thickness of *stratum corneum* is 10–15 μm and that of the viable epidermis is 50–100 μm, the insertion depth of these MNs could potentially reach beyond the epidermis and into the dermis layer.^46^ Since the drug and polymer composition of RIS-IMN-F2 and RIS-IMN-F7 are identical, with the only variation being the casting process, RIS-IMN-F2 was chosen over RIS-IMN-F7 for further studies due to its shorter manufacturing time. Therefore, RIS-IMN-F2 and RIS-IMN-F3 were selected for further investigations.

### 3.4 Differential Scanning Calorimetry Analysis of RIS IMNs

**Figure 4:**
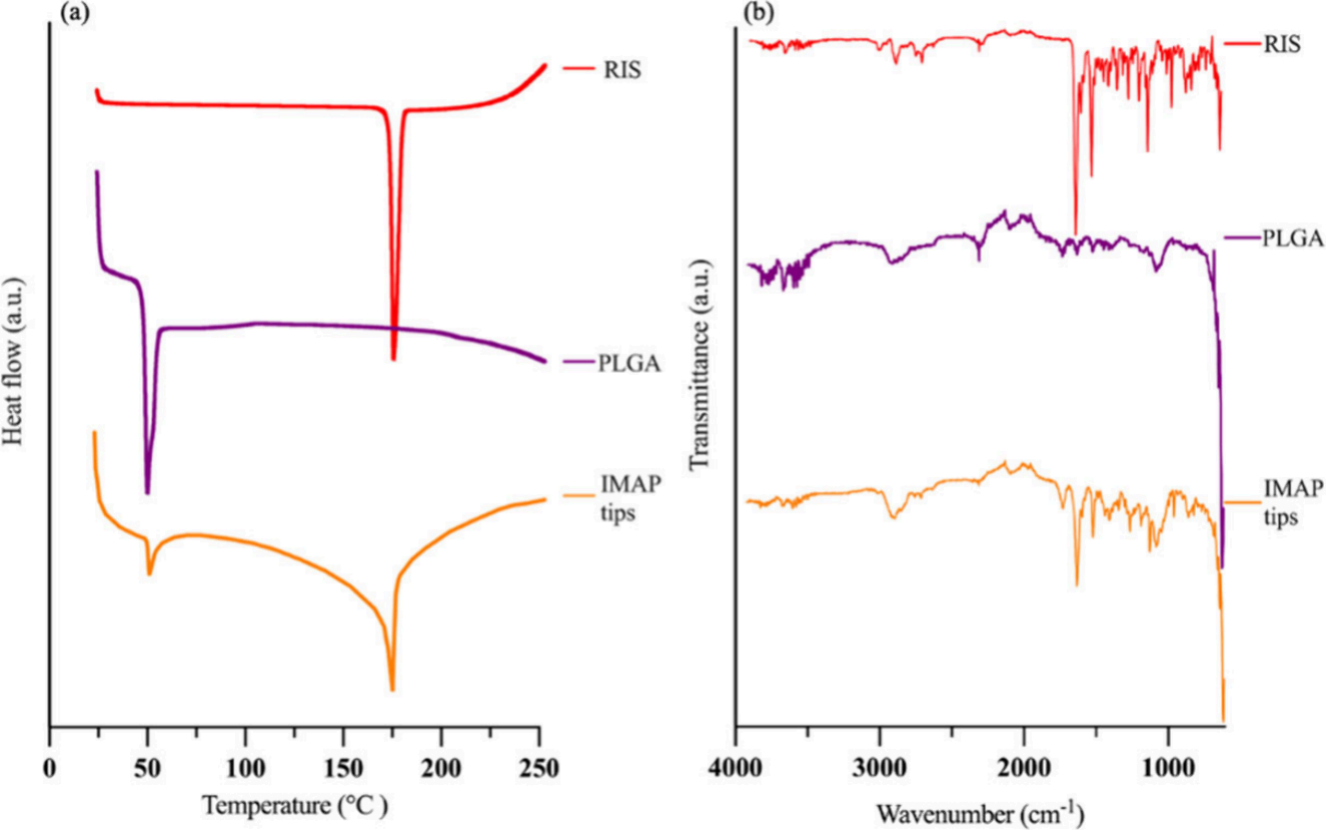
(a) DSC thermograms (exo up) and (b) FTIR spectra of RIS, PLGA, and implantable MN tips.

As can be seen in Figure 4a, a sharp endothermic peak at 171.45 °C was observed for pure RIS, which matches its melting point and also confirmed its crystalline structure.^47^ An endothermic peak at 47.92 °C was also observed for PLGA, which corresponds to its glass-transition temperature.^48^ Both peaks were then observed in the curve of powders obtained from the MN tips of RIS IMNs, indicating that RIS IMNs are formed by a physical mixture of RIS and PLGA and that there is no chemical interaction between them.

### 3.5 Attenuated Total Reflectance Fourier Transform Infrared Analysis of RIS IMNs

FTIR was used to investigate potential chemical interaction between RIS and PLGA within IMNs through the analysis of functional groups present in the drug, polymer, and MNs, the FTIR spectra of which are shown in Figure 4b. For RIS, the weak absorption band at 3044 cm^–1^ was associated with the aromatic C–H stretching vibration, while the aliphatic C–H stretching vibrations appeared at 2937 cm^–1^. A prominent absorption band at 1649 cm^–1^ was attributed to the C=O stretching of the δ-lactam ring. Additionally, the peak at 1534 cm^–1^ resulted from C=C stretching in the aromatic ring. The band at 1191 cm^–1^ was attributed to the C–N stretching vibration from the piperidine ring, and a strong peak at 1130 cm^–1^ corresponded to the aryl fluoride group.^49^ In the MN tips’ spectrum, all these characteristic RIS peaks were observed, confirming that no chemical interaction occurred between the components in the final MN tips.

### 3.6 Saturation Solubility Study of RIS

Before conducting any drug release studies, one of the important considerations is to ensure sink conditions during drug release as the drug release rate will significantly be hindered at concentrations where drugs are rather saturated. A common practice is to achieve skin condition in which drug concentration is at least 3 times lower than the drug saturation concentration.^50^ Therefore, a drug saturation study to test the drug solubility in an aqueous medium with or without the presence of surfactants was also carried out. As presented in Table 4, the solubility of RIS in PBS was 0.22 mg/mL, which was rather low. Since the volume of release media used in this study is 12 mL, and each IMN typically contains more than 1 mg of drug, it is unlikely to meet the sink condition for RIS. Therefore, a variety of surfactants and solvents were used to increase the solubility of RIS. It can be seen in Table 4 that only PBS containing 1% w/v SLS had significantly higher solubility of RIS at 2.73 mg/mL. Other common surfactants like Tween and PEG seem to have little effect on solubility enhancement of RIS. As a result, PBS containing 1% w/v SLS was chosen to be the release medium for the *in vitro* release study and *ex vivo* skin deposition and permeation study of RIS IMNs.

**Table 4:** Saturation Solubility of RIS in PBS with or without Solubility Enhancers (MeanValues ± sd, *n* = 4)

solvents	saturation solubility of RIS (mg/mL)
PBS	0.22 ± 0.00
PBS containing 1% (w/v) Tween 80	0.37 ± 0.00
PBS containing 20% (v/v) methanol	0.38 ± 0.01
PBS containing 1% (v/v) PEG 400	0.43 ± 0.01
PBS containing 1% (v/v) PEG 600	0.40 ± 0.00
PBS containing 1% (w/v) SLS	2.73 ± 0.00

### 3.7 *In Vitro* Release Study of RIS IMNs

The *in vitro* release study is an essential method for evaluating sustained-release drug delivery systems. Previously, the dialysis membrane has been commonly used for *in vitro* drug release from MNs as porcine skin is not suitable for multiple-day drug release. However, dialysis membrane is inherently different from skin, making the results less convincing. In the current study, agarose gel was used as the model skin in combination with a Franz cell setup for the *in vitro* drug release study to better simulate the tissue environment. Agarose gel offers suitable rigidity that more closely simulates the tissue encapsulation of the dosage form, as opposed to traditional drug release for MNs where each MN placed in a dialysis bag freely immersed in aqueous media. Notably, the agarose gel layer acts as a physical barrier that holds the MNs in place but does not impede the drug release from the formulations, as reported in a previous finding.^51^

**Figure 5:**
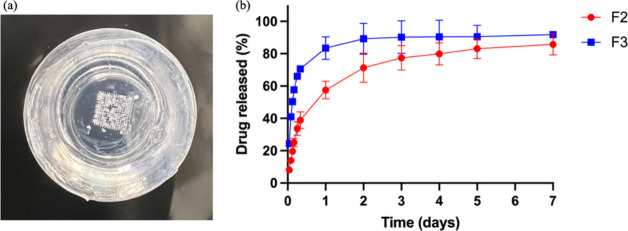
(a) A representative image of agarose gel following the insertion of RIS IMNs. (b) *In vitro* cumulative release profile for RIS-IMN-F2 and RIS-IMN-F3 (mean values ± sd, *n* = 4).

Figure 5a presents the agarose gel following the insertion of RIS IMNs, while Figure 5b presents the cumulative release profiles of RIS-IMN-F2 and RIS-IMN-F3. Both RIS IMN formulations exhibited a similar release pattern, characterized by a relatively rapid drug release within the first 24 h, followed by a slower, sustained release over the following days. After 24 h, RIS-IMN-F2 and RIS-IMN-F3 had released 57.54 ± 5.41% and 83.42 ± 7.03% of the drug, respectively. While RIS-IMN-F3 reached its peak release of 85.66 ± 6.54% on day 3, RIS-IMN-F2 showed a more gradual release, reaching its maximum at 91.84 ± 2.15% on day 7. By day 7, there was no significant difference in the percentage of drug released between the two IMNs (*p* > 0.05). The faster release of RIS-IMN-F3 is likely due to its higher ratio of RIS to PLGA (3:2 w/w), as increased drug loading can accelerate release, a phenomenon noted in previous studies.^52,53^

### 3.8 *Ex Vivo* Skin Deposition and Permeation Study of RIS IMNs

**Figure 6:**
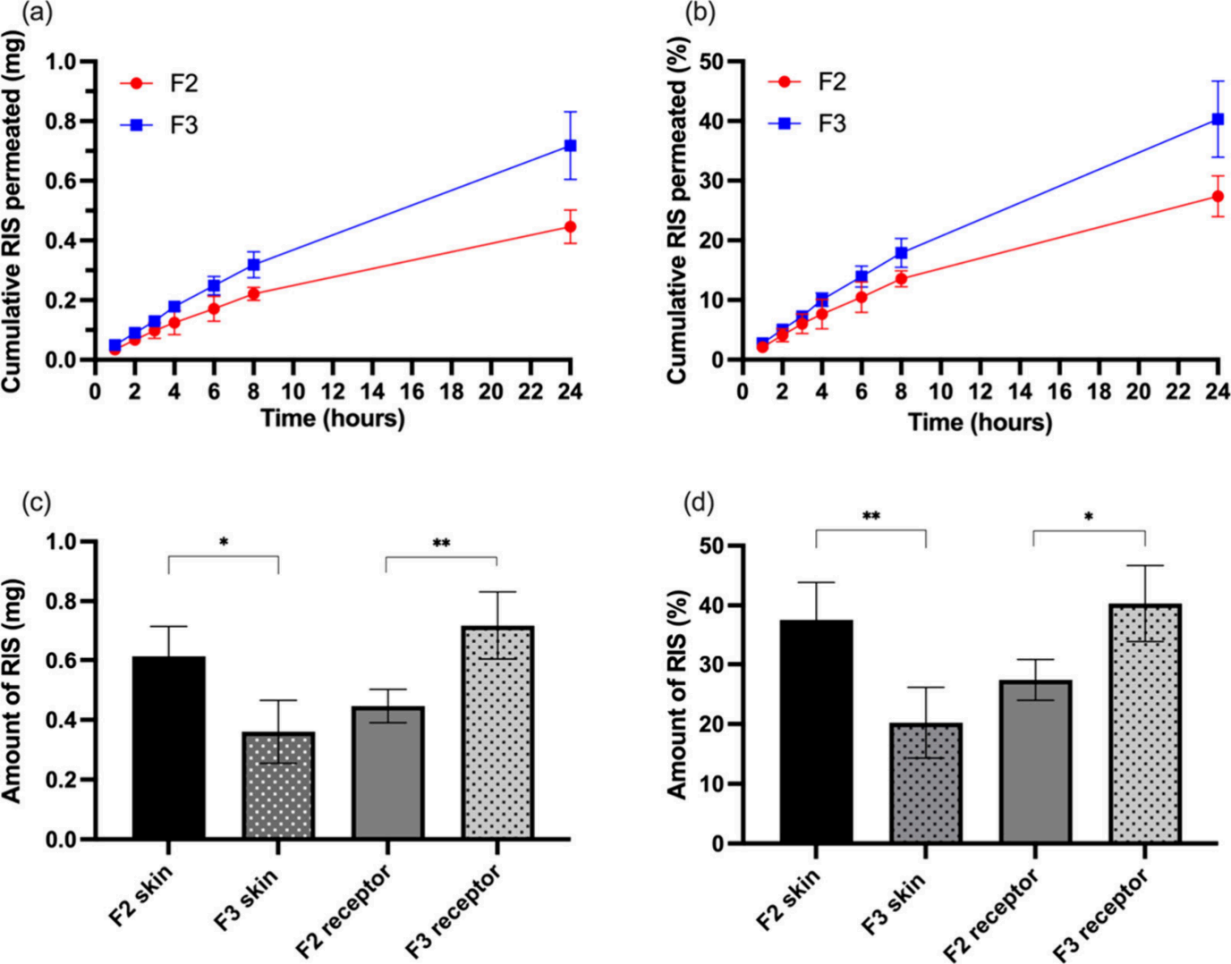
(a) Cumulative permeation of RIS from RIS-IMN-F2 and RIS-IMN-F3 into Franz cells, measured in micrograms over a 24 h period. (b) Cumulative permeation of RIS from RIS-IMN-F2 and RIS-IMN-F3 into Franz cells, expressed as a percentage relative to the drug content in the MNs, over a 24 h period. (c) Amount of RIS deposition in skin and receptor compartment after application of RIS-IMN-F2 and RIS-IMN-F3 for 24 h. (d) Amount of RIS deposition in skin and receptor compartment after application of RIS-IMN-F2 and RIS-IMN-F3 for 24 h, expressed as a percentage relative to the drug content in the MNs (mean values ± sd, *n* = 4; *p < 0.05, **p < 0.01).

The *ex vivo* skin deposition and permeation study were conducted to assess the drug delivery efficiency of this innovative transdermal system using a Franz cell apparatus. Stillborn pig skin was chosen as the model due to its well-documented similarities to human skin, both in structure and permeability.^54,55^ Following the insertion of RIS IMNs into porcine skin, the dissolvable part of the MN tips fabricated from PVP/PVA was first dissolved by the interstitial fluid within the skin, leaving the drug-loaded PLGA MN tips deposited in the skin. As seen in Figure 6a,b, RIS permeation from both RIS-IMN-F2 and RIS-IMN-F3 into the Franz cell increased steadily over time in almost zero-order. At 24 h postapplication, cumulative permeation values for RIS-IMN-F2 and RIS-IMN-F3 were 0.45 ± 0.06 mg and 0.72 ± 0.11 mg, respectively. Figure 6c,d shows that after 24 h, 0.61 ± 0.10 mg of RIS was deposited and still remained in the skin from RIS-IMN-F2, whereas only 0.36 ± 0.11 mg was deposited and still remained in the skin from RIS-IMN-F3, with a statistically significant difference between the two (*p* < 0.05). The overall delivery efficiency, calculated by combining the RIS deposited in the skin and the RIS permeated to the receptor compartment, was 65.03% and 60.67% for RIS-IMN-F2 and RIS-IMN-F3, respectively, as detailed in Table 5. These findings are consistent with our previous research in which PLGA-based IMN achieved over 60% of drug delivery efficiencies.^20^

**Table 5:** Summary of Delivery Efficiency of Two Formulations of RIS IMNs (Mean Values ± sd, *n* = 4)

formulation code	RIS-IMN-F2	RIS-IMN-F3
Drug deposited in skin (mg)	0.61 ± 0.10	0.36 ± 0.11
Drug delivered in receptor (mg)	0.45 ± 0.06	0.72 ± 0.11
Total drug delivered (mg)	1.06 ± 0.08	1.08 ± 0.16
Drug content (mg)	1.63 ± 0.13	1.78 ± 0.34
Delivery efficiency (%)	65.03 ± 4.95	60.67 ± 9.98

The release of the drug from PLGA MN tips undergo two distinct mechanisms as discovered in previous research.^56^ Although PLGA is a hydrophobic polymer, which effectively prevents the loaded cargo inside the tips from escaping, the porous polymer structure may still allow a small amount of incorporated drug to release into the skin and permeate into the receptor compartment of Franz cell. This mimics *in vivo* conditions, where the drug is absorbed into the bloodstream and exerts systemic effects. Second, the PLGA tips gradually degrade over the course of several weeks to several months, releasing the loaded drug simultaneously. However, the PLGA tips are unlikely to degrade by a large amount within 24 h of this experiment; the remaining PLGA tips are still deposited within the skin along with its incorporated RIS, which can be confirmed by the data presented in Table 5. Over 60% of RIS in RIS-IMN-F2 was still retained in the porcine skin after 24 h. In this investigation, RIS-IMN-F2 demonstrated superior drug retention in the skin and a slower release rate compared to that of RIS-IMN-F3, potentially due to its higher PLGA content. The aim of the previous experiments was to demonstrate the feasibility of IMNs to deposit PLGA tips loaded with RIS into the skin and the sustained drug release. It is important to mention, as it will be shown in the *in vivo* experiment section, RIS is metabolized to 9-OH RIS that is an active metabolite extending the activity of the drug.^57^ This is important, as the work presented so far focused only on RIS delivery.

### 3.9 Biocompatibility Studies of RIS IMNs

**Figure 7:**
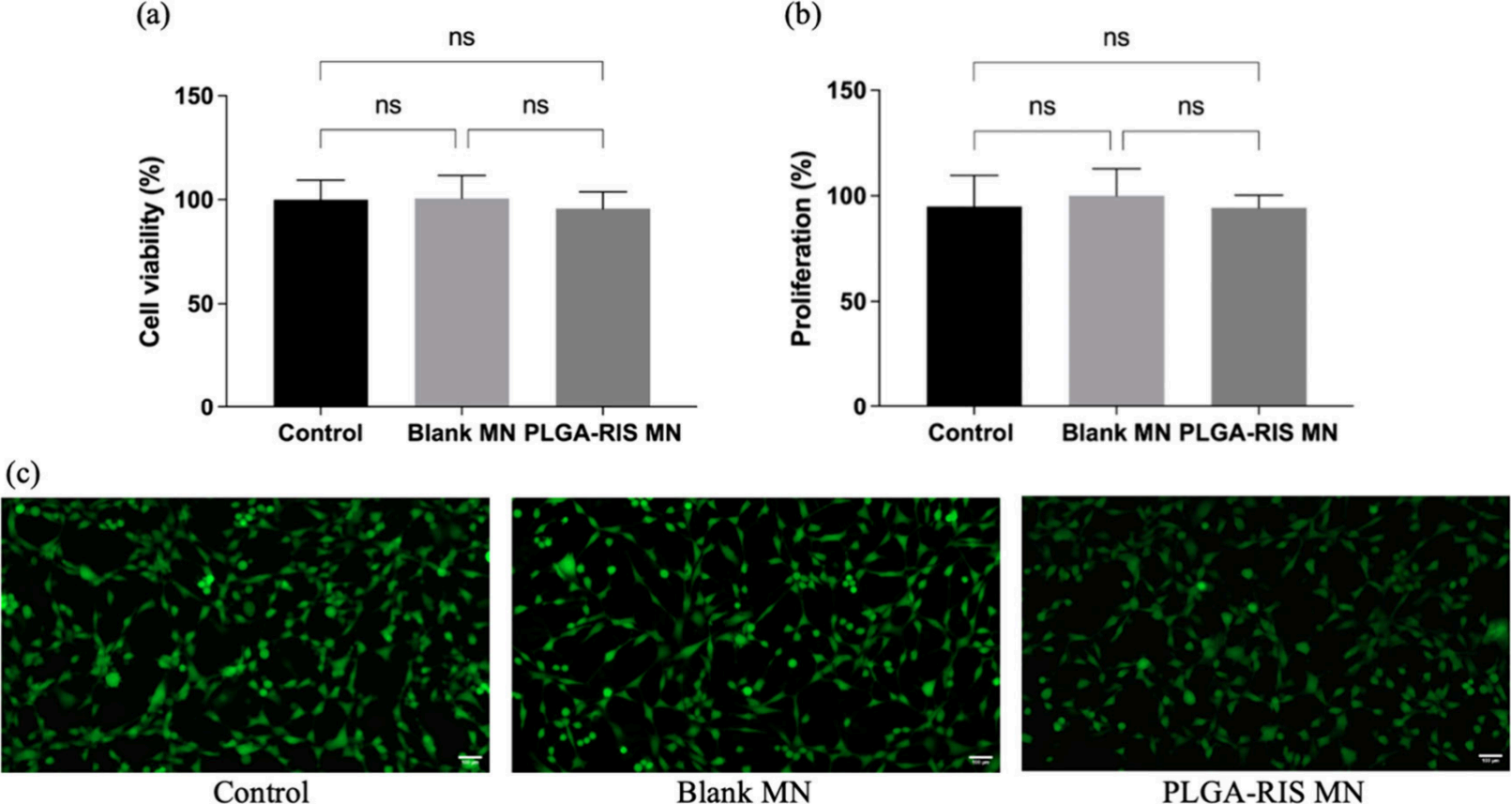
Biocompatibility study. (a) MTT assay at 48 h on formulations of Blank-PLGA-MNs (Blank MN), PLGA-RIS MNs formulation, and cell culture plate (control). (b) Cell proliferation on percentage and (c) live and dead assay (scale bar 100 μm) with plate cells culture (control) and formulations (mean values + sd, *n* = 6).

Biocompatibility is a key requirement for pharmaceutical and medical products like IMNs. It is also well established that *in vitro* toxicity studies are more reliable when cell lines closely resembling the relevant human tissue are used.^58^ Therefore, an *in vitro* biocompatibility test was conducted to assess whether PLGA-based RIS IMNs were biocompatible with human dermal fibroblasts (HDFa). The MTT assay results, shown in Figure 7a, revealed no significant impact on cell viability in HDFa cells after 48 h of exposure to the formulations. These findings suggest that the materials induce a grade 0 cytotoxicity level, based on previously defined cytotoxicity categories.^59^ Similar findings have been reported in other studies, where RIS-loaded MNs were also shown to have no toxic effect on HDFa cells, supporting the current results.^60^ Overall, the data indicate that the formulations are nontoxic to human dermal cells. Additionally, cell proliferation was assessed by measuring the total DNA content, as shown in Figure 7b. Compared to the control group, treatment with both blank IMNs (*p* = 0.7367) and RIS IMNs (*p* = 0.9936) did not result in any significant reduction in cell proliferation. These results are consistent with the cell viability findings. The outcomes from the MTT assay and proliferation test were further corroborated by the live/dead assay (Figure 7c), where live cells and the absence of cell death were clearly observed. These combined results confirm that the formulations exhibit good cytocompatibility, making them promising candidates for use in skin applications.

It is important to note that RIS IMNs were prepared using well-established biocompatible materials approved by the FDA for parenteral use. While the observed results are not unexpected, further testing is needed to assess the *in vivo* toxicity of RIS IMNs. Long-term effects, such as repeated application and inflammation, should also be evaluated.^61^ However, as this is a proof-of-concept study, these objectives fall outside the scope of this work.

### 3.10 *In Vivo* Drug Delivery of RIS IMNs

**Figure 8:**
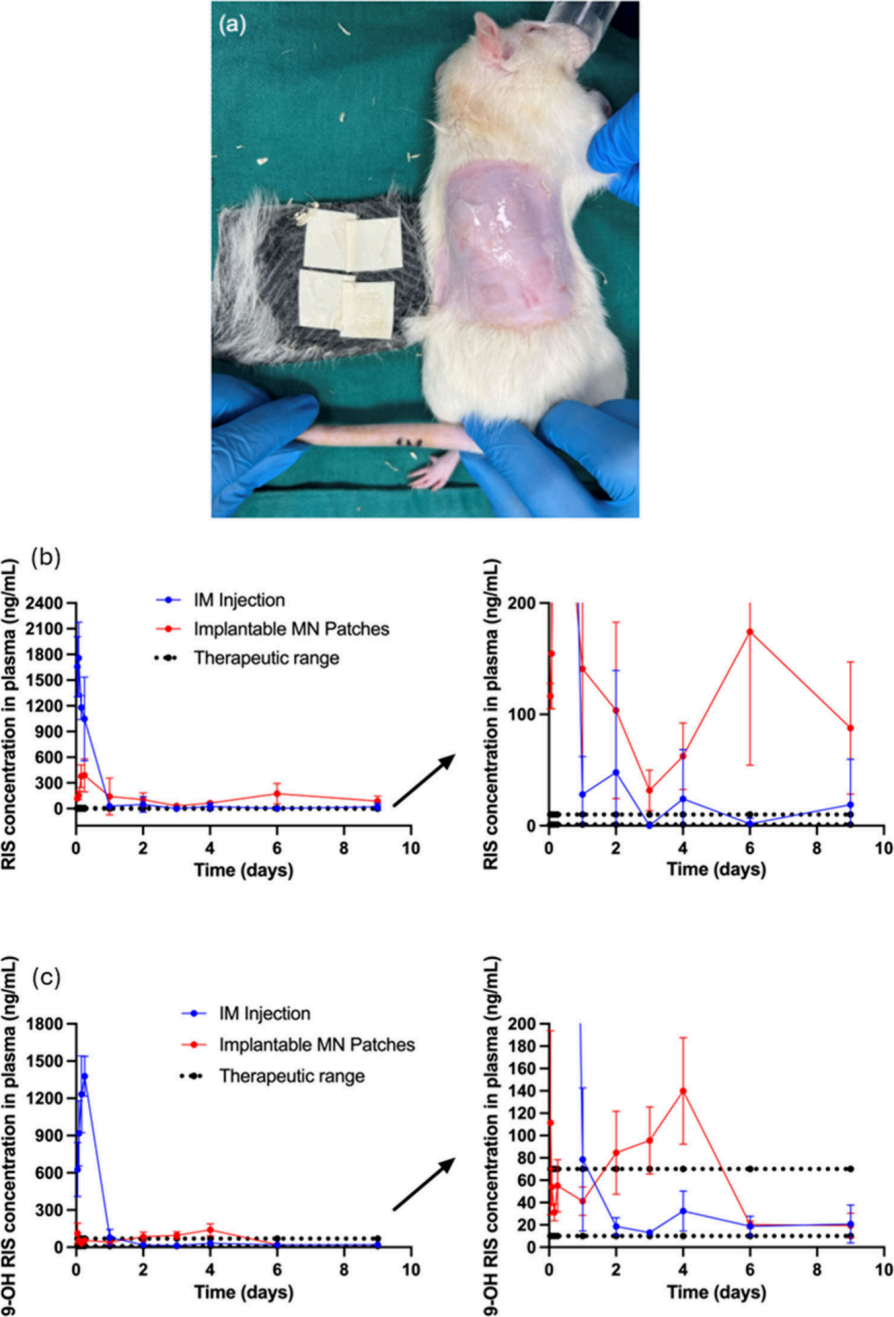
(a) The dorsal area of rat skin upon the removal of RIS IMNs 24 h after application. *In vivo* plasma pharmacokinetic profiles of the rats from IM control cohort, MN cohort following 9 days showing (b) RIS and (c) 9-OH RIS plasma levels (mean values ± sd, *n*  ≥  4 at each time point, according to the experimental regime employed).

An *in vivo* animal study using a female Sprague-Dawley rat model was carried out to get an insight into how RIS IMN would release the drug in living animals. In the actual experiment, each rat received 4 RIS-IMN-F2 containing approximately 6.4 mg of RIS. The delivery efficiency of RIS IMNs in the *ex vivo* study was approximately 60%. Assuming that the bioavailability of IM injection is close to 100%, 100 μL of nanosuspension containing 3.85 mg of RIS was given to each rat intramuscularly in the control group. Due to animal regulations, MNs can only be applied on the rats for no more than 24 h. As a result, all RIS IMNs were removed from the back of the rats 24 h postapplication. As shown in Figure 8a, which shows the dorsal area upon removing the adhesive layer, most of the MNs were implanted, and some residuals of the polymer baseplate were left on the skin or tape. Importantly, no skin abnormalities such as irritation were observed upon the removal of the MN patches.

After entering the systemic circulation, RIS will be metabolized in the liver by enzyme CYP2D6, which converts it into its active metabolite 9-OH RIS, which also possesses therapeutic effect.^57^ A previous study suggested that the therapeutic range of RIS is 1–10 ng/mL while the therapeutic range of 9-OH RIS is 10–70 ng/mL.^62^ Therefore, both compounds were analyzed using LCMS/MS. Figure 8b,c shows the pharmacokinetic profiles for both RIS and its active metabolite 9-OH RIS, from both control and IMN groups. A detailed summary of key pharmacokinetic parameters is also provided in Table 6.

**Table 6:** Pharmacokinetic Parameters of RIS and Its Active Metabolite 9-OH RIS from the IM Control Cohort and the IMN Group^a^

	RIS		9-OH RIS	
pharmacokinetic parameters	IM cohort	IMN cohort	IM cohort	IMN cohort
*C*_max_ (ng/mL)	1756.70 ± 246.06	387.96 ± 194.02	1377.38 ± 160.78	139.89 ± 47.68
*T*_max_ (days)	0.08	0.25	0.25	4
AUC (ng/mL·day)	820.3 ± 212.70	1125.0 ± 286.90	974.3 ± 84.44	537.1 ± 66.65

a Mean values  ±  sd, *n*  ≥  4 at each time point, according to the experimental regime employed.

As shown in Figures 8b, the plasma concentration of RIS rose quickly following the IM injection, reaching its peak concentration (*C*_max_) of 1756.70 ± 246.06 ng/mL at 2 h (*T*_max_). Afterward, the RIS plasma concentration dropped sharply, falling below the analytical method’s LLOQ (41.97 ng/mL) by 24 h. At subsequent time points, the concentrations remained under the LLOQ until the study’s conclusion. The AUC_0–9_ for RIS in the IM group was 820.3 ± 212.70 ng/mL·day. In comparison, the IMN group showed a delayed drug release as can be seen in Figure 8b, with the average RIS plasma concentration peaking at 387.96 ± 194.02 ng/mL at 6 h (*T*_max_). The plasma concentration of RIS in the IMN cohort was consistently above the therapeutic range in the following days until the end of experiment on day 9, when the RIS plasma concentration was detected at 87.78 ± 34.09 ng/mL.^63^ The AUC_0–9_ in the IMN group was 1125.0 ± 286.90 ng/mL·day. A statistically significant difference in peak plasma concentrations was observed between the control and IMN groups (*p* = 0.03).

In this study, 9-OH RIS was detected at the first hour after the application of both IM injection and IMN and then increased until reaching the peak (Figure 8c). The pharmacokinetics profiles demonstrated the rapid absorption and metabolism of RIS to 9-OH RIS. The maximum concentration (*C*_max_) was 1377.38 ± 160.78 ng/mL at 6 h (*T*_max_) postinjection in the IM control cohort, while a maximum concentration (*C*_max_) of 139.89 ± 47.68 ng/mL at 4 days (*T*_max_) postapplication was detected in the IMN cohort. The AUC values of the IM cohort and IMN cohort are 974.3 ± 84.44 and 537.1 ± 66.65 ng/mL·day, respectively. As mentioned earlier, 9-OH RIS is also pharmacologically active and contributes to the overall therapeutic effects, although the metabolism rate can vary significantly between individuals due to genetic differences in CYP2D6 activity.^64^ It has been reported that the half-life for the active fraction of the drug (RIS and 9-OH RIS combination) is around 20 h.^65^

In the present study 9-OH RIS was detected at 626.17 ± 217.84 ng/mL 1 h after the IM injection, with its plasma concentration continuing to rise until reaching the peak (*C*_max_) at 1377.38 ± 160.78 ng/mL at 6 h (*T*_max_). The pharmacokinetic profiles highlighted the rapid absorption and metabolism of RIS into 9-OH RIS. In comparison, the 9-OH RIS plasma concentration in the IMN group was much more stable over the course of the experiment, with the active metabolite still being detected on day 9 at 19.38 ± 11.00 ng/mL. The peak concentration (*C*_max_) of 139.89 ± 47.68 ng/mL occurred 4 days (*T*_max_) after IMN application. The AUC values of 9-OH RIS for the IM and IMN cohorts were 974.3 ± 84.44 and 537.1 ± 66.65 ng/mL·day, respectively. Similar to RIS, the plasma concentration of 9-OH RIS was above the therapeutic range throughout the 9-day experiment, demonstrating much more sustained release of RIS compared with that of traditional IM injection.

Relative bioavailability (*F*) is determined by comparing the AUC of RIS after administration of the IM injection and MN application. In this case, it was calculated using eq 3,
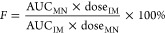
3where dose_IM_ is the drug dose that each rat was given in the IM control cohort, and dose_MN_ is the drug dose that each rat was given in the IMN cohort. AUC_MN_ is the AUC value of plasma from the RIS IMN application, and AUC_IM_ is the AUC value of plasma from RIS IM injection.

Assuming the bioavailability of IM injection of RIS is 100%, the relative bioavailability of RIS from the IMN is calculated to be 49.50%, which is close to bioavailability results in our previous studies and is considered remarkable for transdermal drug delivery as delivery efficiency of conventional transdermal drug delivery systems are typically very low.^22^ For example, less than 20% of inulin was able to be delivered across the skin even with the help of nanocarriers.^66^

This proof-of-concept study demonstrates the capability of IMNs to provide sustained release of RIS. The findings indicate that these devices can maintain RIS levels above therapeutic levels. However, further optimization is necessary, as the observed plasma drug levels exceeded the therapeutic window. It is important to note that these studies were conducted in rats and patch sizes would need to be adjusted for testing in larger animals and humans. Based on these preliminary results, estimations can be made regarding patch size for human applications. However, it must be emphasized that these are only estimations, and clinical trials would be required to evaluate the performance of such patches. Since the recommended dosing of long-acting injectable risperidone is 25–50 mg every 2 weeks and the area of each RIS IMN is 0.36 cm^2^, an IMN size of 11.15–22.31 cm^2^ is enough to deliver RIS for a two-week treatment, which can be done in a single MN application.^67^ It is important to note that applying a larger MN may not be as easy and convenient as applying a single small MN at the laboratory scale. Nevertheless, our previous study, in which human subjects applied larger MNs directly on their bodies, has demonstrated that the insertion depths of large MNs had no significant difference from that of small MNs.^68^ As mentioned earlier, the RIS delivery efficiency is not the only parameter that affects the effectiveness of the treatment. As risperidone metabolite (9-OH RIS) is active, too, patient metabolic activity should be considered. Also, it is important to consider that RIS requires titration for patients suffering from schizophrenia. This can be addressed by preparing patches with different specific sizes. It has been reported before that altering the patch size has a direct effect on drug delivery *in vivo*.^26^ Additionally, this approach has been used for NEUPRO rotigotine patches.^69^

The results suggest that IMNs can provide drug release for approximately 2 weeks, requiring the patient to reapply the IMN after this period. This system does not require invasive injections or healthcare professionals for application, offering significant advantages over injectable long-acting systems, even if the latter provide a longer drug release. The self-application capability makes IMNs particularly suitable for patients with limited access to healthcare professionals such as those living in remote areas.

The IMN also holds unique advantages over other MN systems such as dissolving MN (DMN) and hydrogel-forming MN (HMN) when it comes to the sustained release of long-acting therapeutics like RIS. DMN typically delivers fine drug powder or drug nanocrystals into the skin, which normally experience a relatively quick drug release rate. A previous DMN system loaded with RIS nanocrystals had immediate release of most drugs within the first 2 days with both RIS and 9-OH RIS plasma concentrations quickly dropping to therapeutically irrelevant levels thereafter.^60^ On the other hand, HMN is known for being able to deliver a large number of hydrophilic drug molecules via its tailorable drug reservoir. However, when encapsulating hydrophobic drugs into solubility enhancer, they may also be delivered with HMN. For instance, RIS was previously encapsulated into cyclodextrin and formulated into a drug reservoir, which was used as a part of HMN system.^70^ Although RIS was successfully delivered through the highly swollen hydrogel network with the help of cyclodextrin, most of the drug was again absorbed into the systemic circulation within the first few days with no sustained release of RIS. In contrast, IMNs deliver drugs of interest by planting biodegradable PLGA MN tips in the skin, which will gradually degrade and release encapsulated cargos. Importantly, one could always optimize the drug release rate by using a certain type of PLGA material. In our case, both RIS and 9-OH RIS drug plasma concentrations were relatively stable over the course of the entire experiment, demonstrating that it is probably a better option for MN-based transdermal delivery of RIS.

## 4 Conclusion

In an attempt to tackle the increasing need for self-administered treatments for mental health conditions such as schizophrenia, this study successfully designed and fabricated an IMN loaded with RIS, one of the most used antipsychotic medications. These IMNs exhibited a well-structured design and strong mechanical strength, enabling them to penetrate the tough skin barrier. *In vitro* skin deposition and permeation study demonstrated that the RIS IMN could potentially achieve over 65% drug delivery efficiency, which is much higher than conventional transdermal drug delivery technologies. *In vivo* animal study using Sprague-Dawley rats showed that the RIS IMN enables a much more prolonged release of RIS from its PLGA tips, offering extended drug release over a week, compared with IM injection of RIS nanosuspensions which had therapeutic effect for merely 24 h as well as previously developed RIS DMN and HMN which quickly release drug into systemic circulation and which could only maintain therapeutic drug plasma for less than 3–5 days. Thus, this RIS IMN presents a promising value proposition in schizophrenia treatment by offering a self-applicable skin patch that can potentially provide therapeutic effect for 2 weeks with one single application.
