# Correction to “Schizophrenia Treatment Based on Sustained Release of Risperidone from Poly(lactic-*co*-glycolic) Acid Implantable Microarray Patch “

**DOI:** 10.1021/acsami.5c20098

**Published:** 2025-10-21

**Authors:** Linlin Li, Li Zhao, MingShan Li, Yushi Tao, Akmal Hidayat Bin Sabri, Natalia Moreno-Castellanos, Rand Ghanma, Brett Greer, Qonita Kurnia Anjani, Helen O. McCarthy, Ryan F. Donnelly, Eneko Larrañeta

The authors regret that an error occurred during the preparation of Figure 7c in the originally published version of this article. The live/dead cell images included do not correspond to the studies described. The correct live/dead images are provided below.

**Figure 7:**
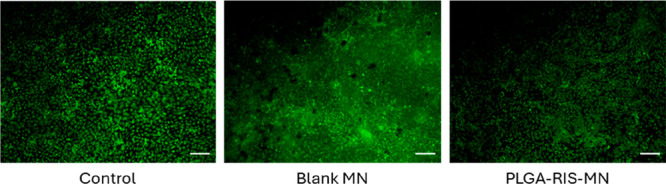
(c) Live and dead assay (scale bar 100 μm) with plate cells culture (control) and formulations

This correction does not affect the description, interpretation, or conclusions of the study. The live/dead images were included for illustrative purposes only, as cytocompatibility was assessed using the MTT assay. The authors apologize for any inconvenience this may have caused.

